# Thermophysical and Mechanical Properties of Granite and Its Effects on Borehole Stability in High Temperature and Three-Dimensional Stress

**DOI:** 10.1155/2014/650683

**Published:** 2014-03-20

**Authors:** Wang Yu, Liu Bao-lin, Zhu Hai-yan, Yan Chuan-liang, Li Zhi-jun, Wang Zhi-qiao

**Affiliations:** ^1^Key Laboratory on Deep Geo-Drilling Technology of the Ministry of Land and Resources, China University of Geosciences, Beijing 100083, China; ^2^State Key Laboratory of Oil & Gas Reservoir Geology and Exploitation, Southwest Petroleum University, Chengdu 610500, China; ^3^State Key Laboratory of Petroleum Resource and Prospecting, China University of Petroleum, Beijing 102249, China

## Abstract

When exploiting the deep resources, the surrounding rock readily undergoes the hole shrinkage, borehole collapse, and loss of circulation under high temperature and high pressure. A series of experiments were conducted to discuss the compressional wave velocity, triaxial strength, and permeability of granite cored from 3500 meters borehole under high temperature and three-dimensional stress. In light of the coupling of temperature, fluid, and stress, we get the thermo-fluid-solid model and governing equation. ANSYS-APDL was also used to stimulate the temperature influence on elastic modulus, Poisson ratio, uniaxial compressive strength, and permeability. In light of the results, we establish a temperature-fluid-stress model to illustrate the granite's stability. The compressional wave velocity and elastic modulus, decrease as the temperature rises, while poisson ratio and permeability of granite increase. The threshold pressure and temperature are 15 MPa and 200°C, respectively. The temperature affects the fracture pressure more than the collapse pressure, but both parameters rise with the increase of temperature. The coupling of thermo-fluid-solid, greatly impacting the borehole stability, proves to be a good method to analyze similar problems of other formations.

## 1. Introduction

Deep resources such as oil, gas, and solid mineral have drawn more interest. Generally, the deeper drill is characterized by higher pressure and temperature, which make the drilling and borehole stability harder [1–7]. However, in Mexico Bay, North Sea Basin, Sichuan Basin, and the South Sea of China [8], for example, the gas and oil reservoirs in layers over 200°C have been successfully exploited.

When the fluid circles, the upper surrounding rock will be heated; when the fluid ceases to work, however, the lower one will be heated. Balanced by the fluid column pressure and the rock confining pressure [9, 10], the heated rock will fail to expand, generating thermal stress as a result [11]. Maury and Guenot claim that the thermal stress contributes most to the instability of the borehole [12]. The outcome they obtained shows that when the temperature of the midhard rock rises up by 1 centigrade, the stress can increase by 0.4 MPa, up to 1 MPa for the harder rock as a result. The thermal stress in 25 MPa to 50 MPa is practically common in 4000 meters boreholes. Consequently, the initial borehole stress and the common thermal stress can work together leading to collapse and fracture.

Wang et al.'s research [13] shows that the Westerly granite can generate thermal cracking when heated up to 75°C. And the threshold value of 60~70°C is suggested by Chen et al. [14]. Impacted by hydrostatic stress and thermal cracking, the granite's peak of the permeability, up to 3.5 × 10~4 mD/°C, to the initial one reaches up to 93 [15]. This indicates that a field characterized by high permeability is developed around the borehole, triggering another stress field. In the borehole, the initial stress, temperature, and the stress field were triggered by overlapping the fluid together, which led to the deformation instability and leakage [9, 16, 17]. Consequently, the instability may make the drill stick or damage the casing.

Since the 1980s, in order to dispose the permanent nuclear waste, people began researching the coupling of THM (thermo-hydro-mechanical) [18, 19]. A global International cooperation project named DECOVLEX was established in 1992. Since then, a series of experiments, including modeling, have been conducted and some invaluable outcomes have been obtained as a result [20–24]. At the fourth stage of this project, the aim mainly was to study the mechanics of crystalline rock and the process in which the mechanical and hydraulic properties of the EDZ (excavation damage zone) are transformed. This process can harden or soften the rock [25]. In this paper, the thermal physical and mechanical properties of the granite are developed and researched under high temperature and three-dimensional stress. By utilizing* ANASYS-APDL *(ANSYS Parameter Design Language-APDL) [26, 27], the dynamic evolution equations of elastic modulus, Poisson ratio, uniaxial compressive strength, and permeability of granite with temperature are built and run. The temperature-fluid-stress coupling model to analyze the granite's stability is established and simulated to figure out the temperature's influence on collapse pressure, fracture pressure, and stress near the borehole, which can provide theoretical guidance for borehole stability and safety drilling in granite formations.

## 2. Thermophysical and Mechanical Properties

### 2.1. Overview of Experiment

**Figure 1:**
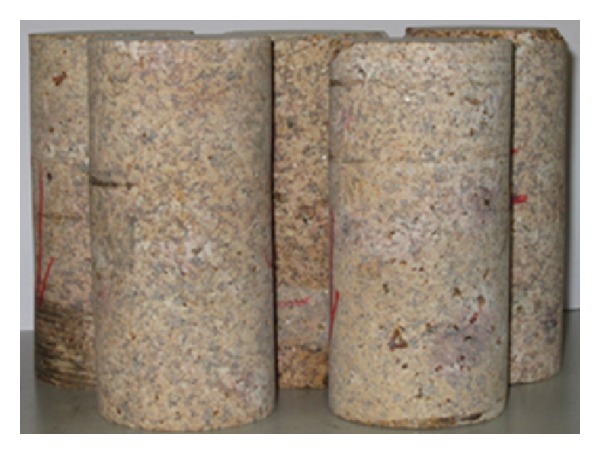
Granite samples for testing.

The sample, obtained from a 1000 meter deep borehole in Mount Yan, North China, is about 100 mm with a diameter of 50 mm. The density is about 2.54 g/cm^3^. TAW-1000 deep pore pressure servo experimental system was employed to test the sample. It consists of quartz, feldspar, and hornblende. All the samples were processed on the basis of Chinese national standard of GB50128-94 (shown in Figure 1). In order to avoid being contaminated by the hydraulic oil, we encapsulated the sample with a 3 mm thickness hot pyrocondensation pipe.

**Figure 2:**
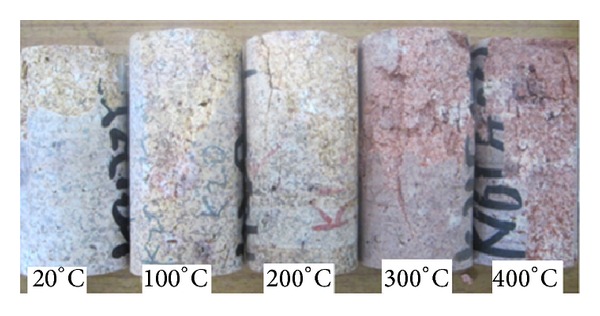
Rock samples correlation under different temperature.

The experiments were conducted in a 1000°C electrothermal furnace whose space is 300 × 200 × 120 mm. The samples were placed at the center of the furnace, to whose front and rear it is about 3 mm far from the sample. All the samples were divided into 5 groups, with each was heated to room temperature, 100°C, 200°C, 300°C, 400°C and insulated for 2 hours, respectively. Compared with the original sample in Figure 2, these heated to 300°C and 400°C is dark red, owing to the Fe^3+^ transformed from Fe.

### 2.2. Longitudinal Wave Velocity Characteristics

**Figure 3:**
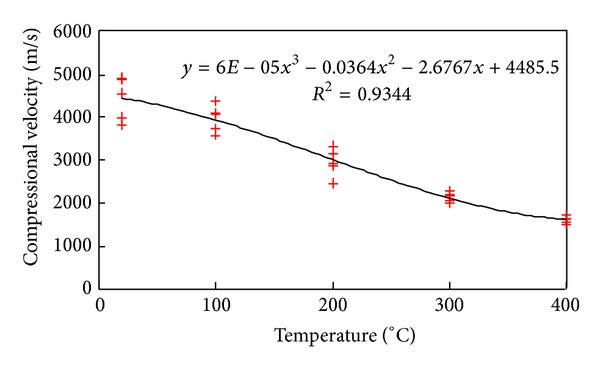
Longitudinal wave velocity variation curve with temperature in granite.

Figure 3 plots the link between longitudinal wave velocity and the temperature. The curve shows that the speed varies inversely with the temperature. This can be accounted for as follows: (I) as free water inside the rock evaporates, the pore becomes bigger; (II) when the temperature increases, the thermal stress will be triggered between minerals, due to their different coefficients of thermal expansion and anisotropy, generating new fractures or expanding the old.

### 2.3. Uniaxial Compression Tests

#### 2.3.1. Uniaxial Strength and Strain

**Figure 4:**
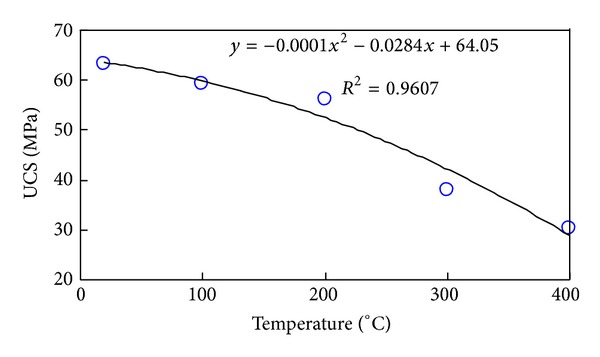
Uniaxial strength variation curve with temperature in granite.

**Figure 5:**
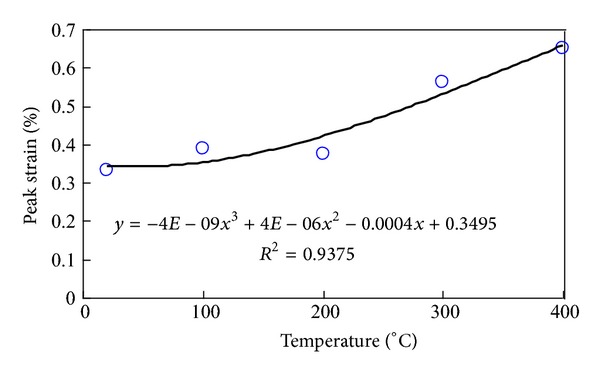
Peak strain variation curve with temperature in granite.

Figure 4 plots the link between temperature and uniaxial strength. It shows that the threshold temperature is 200°C, in accordance with the result obtained from Figure 5. Below 200°C, the sample mainly undergoes brittle fracture, specially divided into compacting and linear elastic phases. On the other hand, over 400°C, the sample mainly undergoes the shear and tensile fractures.

Below 200 centigrade, the peak stress increases slowly but rapidly when it is over 200 centigrade. It shows that the threshold temperature is 200 centigrade, which accords with the outcome obtained from the link between the temperature and the uniaxial strength.

#### 2.3.2. Elasticity Parameters of the Sample

The thermal damage is introduced to reflect the fluctuation of the elastic modulus of the samples before and after the heating the sample. The thermal stress will be produced between different mineral compositions due to the temperature change [28]. The thermal damage is calculated as follows:

(1) $$\begin{array}{c} D\left(T\right)=1-\frac{E_{\left(T\right)}}{E_{\left(0\right)}}. \end{array}$$

The elastic modulus decreases with the increase of the temperature. Additionally, *e* the relationship between the elastic modulus and temperature is fitted by the data, and its fitting formula is *E* = −0.0145*T* + 29.997, with a goodness of 0.955.

**Figure 6:**
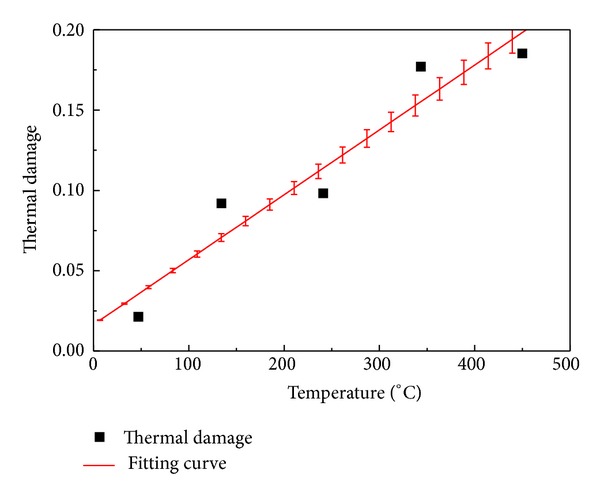
Thermal damage curve under different temperatures in granite.

Figure 6 displays the increase of thermal damage after the sample was heated. As same as aforementioned, the threshold temperature obtained from the* D*-*T* curve is also 200 centigrade. When the temperature is from 0 centigrade to 100 centigrade and over 200 centigrade, the thermal damage of the sample is increasing while the thermal damage is unchanged from 100 centigrade to 200 centigrade.

**Figure 7:**
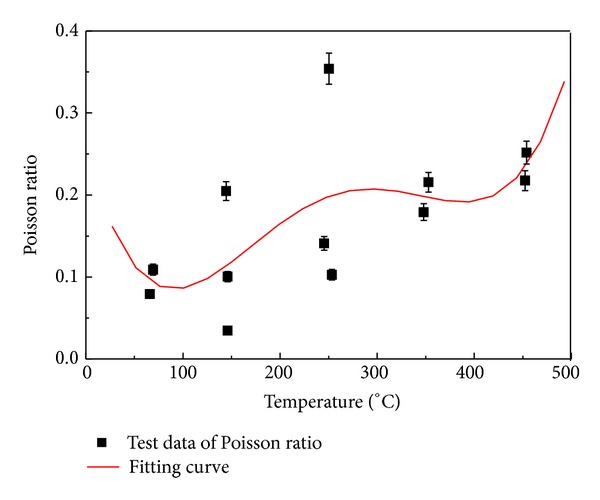
Poisson ratio curve under different temperatures in granite.

The Poisson ratio is characterized by polymeric. As shown in Figure 7, with the increasing of the temperature, the Poisson ratio of the granite samples is more and more mounting. The proportion between Poisson ratio and temperature is mainly accounted for two reasons: (I) the increase of the temperature leads to the changes of the sample's interior structure, the water content, and the porosity; (II) and the temperature and stress are beyond the sample's elasticity.

#### 2.3.3. Damage States of Samples

**Figure 8:**
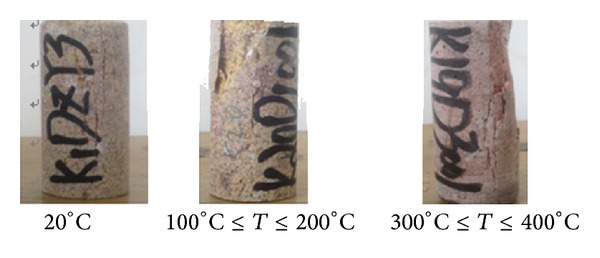
Ordinary damage states under uniaxial pressure.

The sample was experimentally damaged under uniaxial pressure in three ways as shown in Figure 8: (I) under room temperature, the sample undergoes the brittle fractures developing along the axial direction. (II) Under 100–200°C, the sample undergoes the shear fracture. If loaded, the softer part would be damaged without losing its bearing capacity. (III) Under 300–400°C, being sheared and tensioned, the sample undergoes the column fractures.

### 2.4. Triaxial Compression Tests

#### 2.4.1. Mechanical Properties with Different Confining Pressure

**Figure 9:**
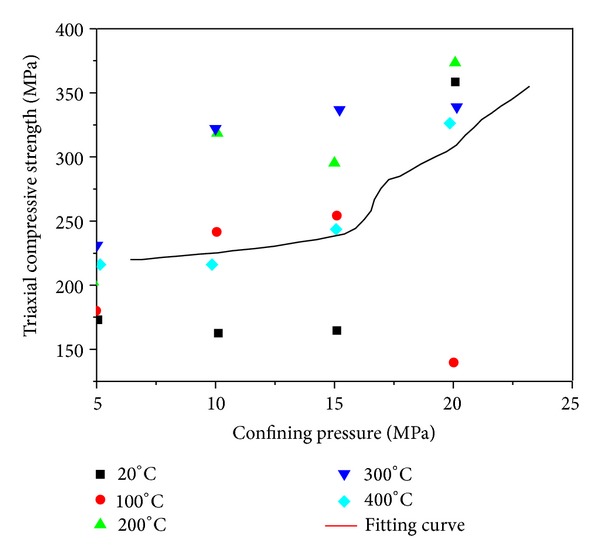
Triaxial compressive strength curve with confining pressure and temperature.

Figure 9 displays the link between triaxial compressive strength and confining pressure. It shows that as the confining pressure rises, the triaxial compressive strength virtually and nonlinearly increases. With *R* = 0.996, the nonlinear link can be expressed as

(2) $$\begin{array}{c} \sigma_{s}=0.834\sigma_{w}^{2}-14.05\sigma_{w}+269.65. \end{array}$$

**Figure 10:**
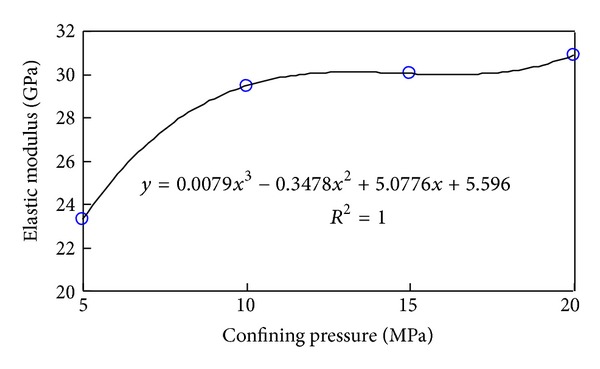
Relationship between elastic modulus and confining pressure under 300°C.

The link between elastic modulus and confining pressure was displayed in Figure 10. The elastic modulus changed the same as the confining pressure except 20 MPa. The threshold pressure is 15 MPa. The elastic modulus can be expressed as

(3) $$\begin{array}{c} E=-0.095\sigma_{w}^{2}+3.085\sigma_{w}+8.775. \end{array}$$

#### 2.4.2. Mechanical Properties with Different Temperatures

**Figure 11:**
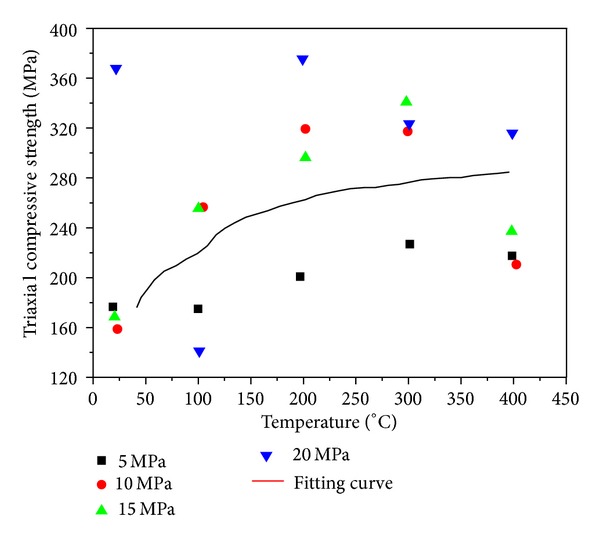
Relationship between triaxial compressive strength and temperature with constant confining pressure.

**Figure 12:**
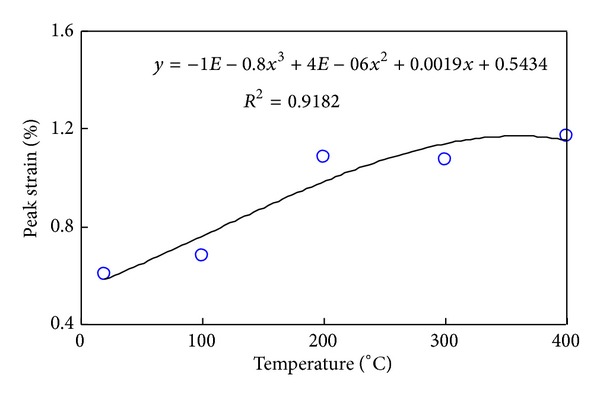
Peak strain variation curve with temperature with constant confining pressure.

**Figure 13:**
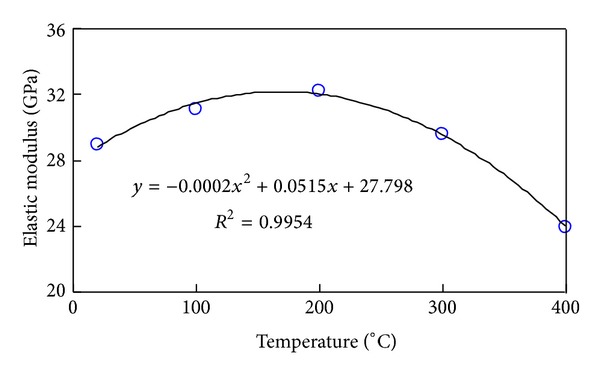
Elastic modulus variation with temperature with constant confining pressure.

Figures 11, 12, and 13, respectively, present the influence exerted by a given temperature on triaxial compressive strength, axial strain at failure, and elastic modulus, which are characterized by discreteness. The three figures confirm that 200°C is the threshold temperature and pressure.

#### 2.4.3. Damage States of Samples

**Figure 14:**
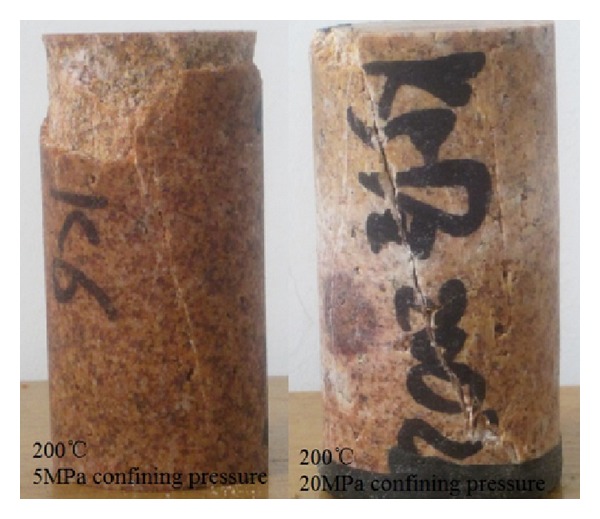
Ordinary damage states under triaxial stress.

Tested by deep pore pressure servo experimental system, the samples were broken by two ways: (I) when heated to 200°C or lower, the sample undergoes the brittle fracture. However, when the confining pressure increased to 20 MPa, the shear and tension fracture dominated. (II) When heated over 200°C, the sample undergoes the compression shear and fracture (Figure 14).

### 2.5. Permeability Effected by Temperature

**Figure 15:**
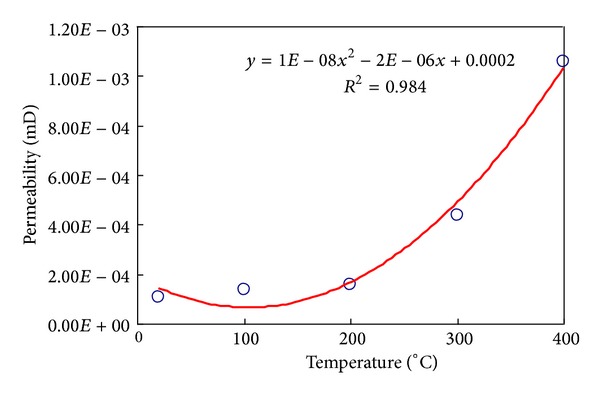
Permeability curve under different temperatures in granite.

The permeability was measured by TAW-1000 deep pore pressure servo experimental system. The sample was enwrapped by a 3 mm thickness hot pyrocondensation pipe. Pressed around by 20 MPa, the sample's one end was ventilated by N_2_ and a highly precise gas flowmeter was installed at its other end.  Figure 15 indicates that the threshold temperature is 200°C. The thermal fracture improves the permeability.

## 3. Finite Element Simulation and Experiment

### 3.1. Basic Equations

Adopting the definition of Biot's effective stress, the relationship between effective stress and total stress is

(4) $$\begin{array}{c} \sigma^{\prime }=\sigma +p_{w}I. \end{array}$$

The mass conservation equation of fluid is

(5) ∂∂xi[ρ1k1ijμ1(∂p1∂xj+ρ1gj)+ρ1k1Tij∂T∂xj]  =n∂p1∂t+ρ1dndt.

The energy conservation equation of solid is

(6) $$\begin{array}{c} \frac{\partial }{\partial t}\left[\left(1-n\right)\rho_{s}\cdot C_{s}\cdot \Delta T\right]=-\frac{\partial q_{si}}{\partial x_{j}}+Q_{s}. \end{array}$$

The energy conservation equation of fluid is

(7) ∂∂t=n·ρ1·C1·ΔT=−∂∂xj(q1i+q1ic),q1i=−λ1ijn∂T∂xjq1i,q1ic=ρ1·C1·ΔT·ν1ir=−ρ1·C1·ΔT·[k1ijμ1(∂p1∂xj+ρ1·gj)+k1Tij∂T∂xj].

Assume that at any point inside the solid phase and liquid phase has the same temperature, the total energy conservation equation [29] can be expressed as

(8) ∂∂t[(1−n)ρs·Cs·ΔT+n·ρ1·C1·ΔT]  =−∂qsi∂xj(qmi+q1ic)+Qs.

The total heat flux density of rock and fluid can be expressed as

(9) $$\begin{array}{c} q_{mi}=q_{si}+q_{1i}=-\left[\lambda_{sij}\cdot \left(1-n\right)+\lambda_{1ij}\cdot n\right]\cdot \frac{\partial T}{\partial x_{j}}. \end{array}$$

Based on mixture theory, the equivalent thermal conductivity can be defined; namely,

(10) $$\begin{array}{c} \lambda_{mij}=\lambda_{sij}\cdot \left(1-n\right)+\lambda_{1ij}\cdot n. \end{array}$$

According to the principle of virtual displacement, the whole equilibrium differential equations in solution domain can be represented as

(11) $$\begin{array}{c} \int_{\Omega }\delta \cdot \varepsilon^{T}\cdot \sigma^{\prime }\cdot d\Omega -\int_{\Omega }\delta \cdot u^{T}\cdot b\cdot d\Omega -\int_{\Omega }\delta \cdot u^{T}\cdot t\cdot ds=0. \end{array}$$

We take the effective stress of rock skeleton equation into (11), and according to the mass conservation equation of the fluid and fluid-solid overall energy conservation equation to form the control equations under the heat-flow-solid coupling. The finite element discretization method can be used to solve the equations' system after it has been transferred to the equivalent credits' weak formulation.

### 3.2. Dynamic Evolution Equations

Based on indoor experiment of this study paper, it was found that the dynamic evolution equations of elastic modulus, Poisson ratio, uniaxial compressive strength, and permeability of Granite with temperature can be represented as

(12) $$\begin{array}{c} E=-0.0145T+29.977,\\ \upsilon =0.0004T+0.1185,\\ \text{UCS}=-0.0001T^{2}-0.0284T+64.05,\\ K=1E-08T^{2}-2E-06T+0.0002. \end{array}$$

### 3.3. Engineering Application Example

The paper uses the ANSYS secondary development function of the fluid-solid interaction module and temperature-structure coupling calculation module for the solver, according to the decoupling method; firstly we do numerical calculation of the granite-borehole temperature field and then put the results into ANSYS fluid-solid interaction of calculation module.

The units' segmentation of temperature field and the units' segmentation of flow-solid coupling calculation is the same, such that the plane-strain problems use four-node units. The dynamic evolution of elastic modulus, Poisson ratio, uniaxial compressive strength, and permeability of granite using secondary development of the ANSYS parametric design language (ANSYS Parameter Design Language-APDL) to achieve. Firstly, to extract the temperature of the unit in the process of thermal analysis calculation, and modify the unit parameters of the thermal and mechanical properties, to form Loop iteration control process, and realize the Granite-borehole temperature coupling.

**Figure 16:**
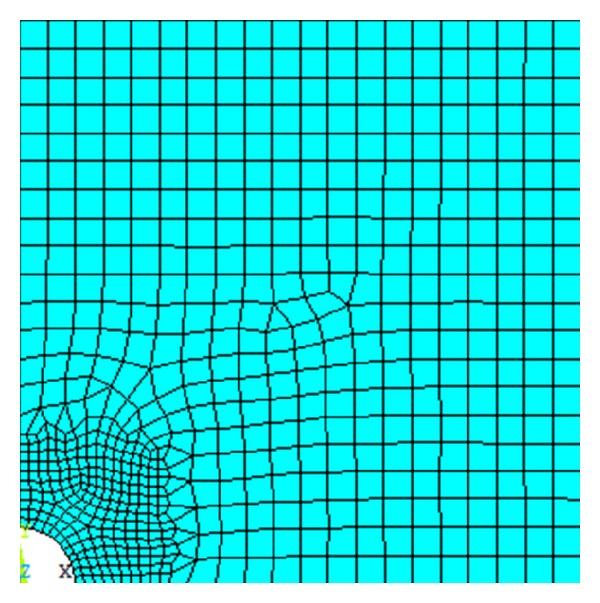
Plane model of borehole.

**Figure 17:**
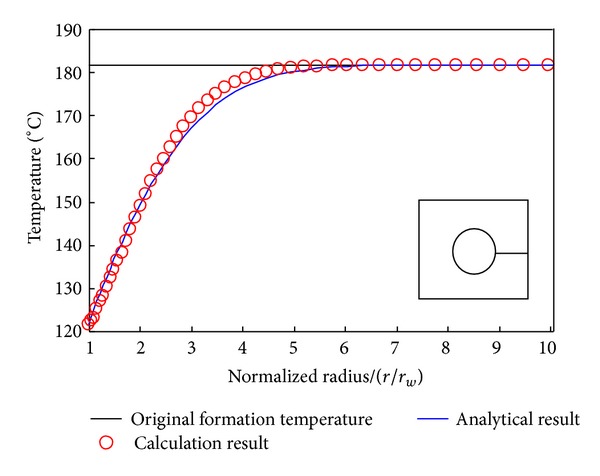
Temperature distribution near borehole.

The stimulation was performed on a one-fourth sample of symmetry. The sample was divided into 612 four-point units in Figure 16. We compared the finite element calculation results with the analytical solutions of Marshall and Bentsen [30] to verify the reliability of the model adopted in this paper. According to the relationship of the rock mechanics of granite that tested indoor and confining pressure, then converted it to the parameters under the condition of confining pressure in this area and applied it to this model, finally the temperature distribution of the borehole surrounding rock was acquired.  Figure 17 illustrated the temperature distribution of the borehole surrounding rock after 8-hour drilling, where the finite element calculation results matched borehole with the analytical solutions. The of the wall and surrounding rock decreased gradually along with the decreasing of drilling fluid temperature, besides the thermal stress of the wall down to the minimum. With the increasing of distance from the borehole, the formation temperature increased gradually until it reached the original formation temperature. The formation that far away from the wall approximately equal five times the boreholebore radius, its internal temperature almost no change, and stayed at the original formation temperature 182°C.

The influence that impacted the granite strata borehole wall stability in the temperature field, the stress field, and the seepage field mainly was exerted by changing the stress state of the borehole [31]. As a result, the original formation of equilibrium was destroyed so that the stress concentration produced around the borehole easily brought up the sidewall instability. The below three kinds of conditions were accounted for to explain the influence on the sidewall stress brought by interconnection. Firstly, do not consider the interconnection of the temperature field and the stress field but consider the interconnection of the seepage field and the stress field. Secondly, do not consider the interconnection of the seepage field and the stress field, but consider the interconnection of the temperature field and the stress field. Thirdly, consider the interconnection of the temperature field and the stress field and the seepage field simultaneously.

#### 3.3.1. Stress in Borehole

**Figure 18:**
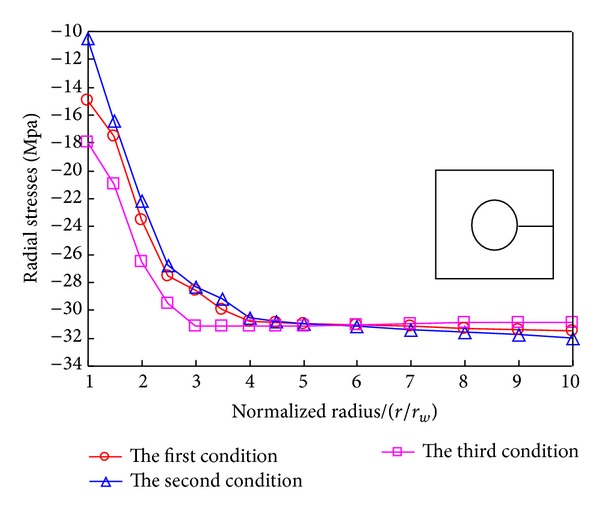
Distribution of the radial stress in borehole under different conditions.

**Figure 19:**
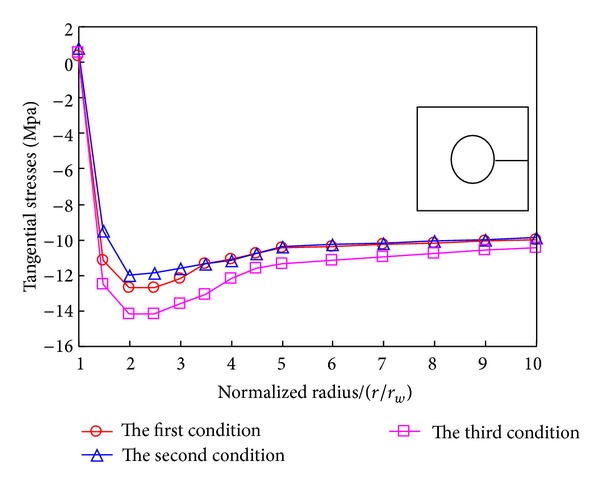
Distribution of the tangential stress in borehole under different conditions.

Figures 18 and 19 display the distribution of radial and tangential stresses peripheral to the borehole. It indicates that temperature and percolation accordantly influence the stress. The minimum stress occurs near the borehole; on the other hand, the samples virtually undergo the same stress under the above three conditions in the further field.

#### 3.3.2. Borehole Stability Effected by Temperature

The shear fracture of the rock, subject to Mohr-Coulomb, expressed by the main stress is described as

(13) $$\begin{array}{c} \sigma_{1}=\sigma_{3}\text{ta}{\text{n}}^{2}\left(\frac{\pi }{4}+\frac{\varphi }{2}\right)+2C\tan\left(\frac{\pi }{4}+\frac{\varphi }{2}\right). \end{array}$$

The shear fracture will occur when the maximum and minimum effective principal stresses are beyond the breaking strength of the rock.

The layer will collapse when the tangential effective stress is over the tensile strength of the rock:

(14) $$\begin{array}{c} \sigma_{\theta }-\alpha P_{P}=-S_{t}. \end{array}$$

The stress distribution is calculated on the basis of finite element. Considering the shear failure and tensile failure, the collapse pressure and tensile pressure are calculated. Suppose the uniaxial compressive strength is subject to temperature. Based on Griffith,

(15) $$\begin{array}{c} \sigma_{c}=\left(8\sim 12\right)S_{t}. \end{array}$$

**Figure 20:**
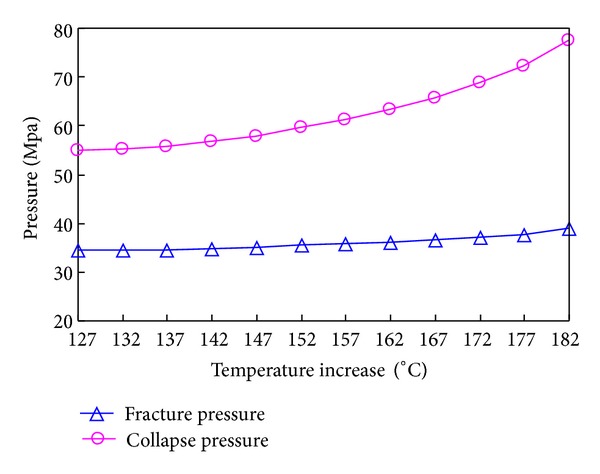
Variation of collapse pressure and fracture pressure with temperature increase.

**Figure 21:**
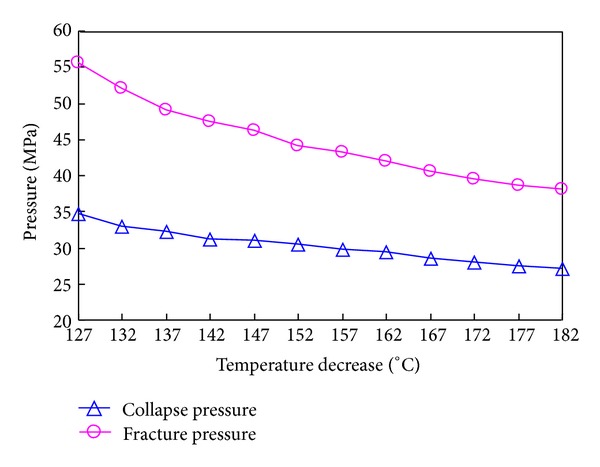
Variation of collapse pressure and fracture pressure with temperature decrease.

The variations of collapse pressure and fracture pressure with temperature increase and decrease are shown in Figures 20 and 21, respectively.

#### 3.3.3. Borehole Stability Affected by Permeability

A filter cake can be developed as the fluid seeps through the permeable reservoir. In this case where the fluid will be constrained, the pore pressure is not equal to the drilling fluid column pressure.

**Figure 22:**
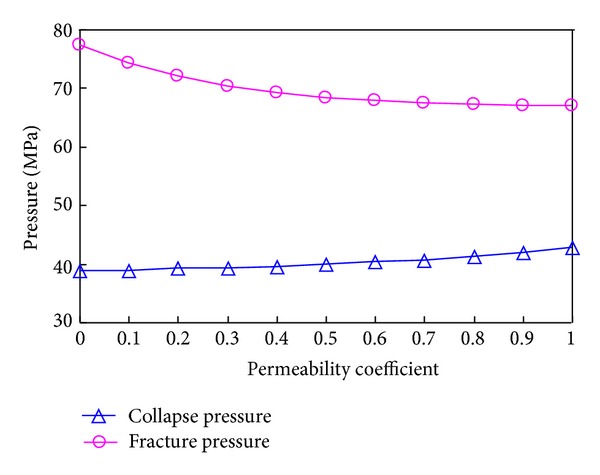
Variation of collapse pressure and fracture pressure with permeability.

Figure 22 plots the link between the permeability coefficient and the collapse and fracture pressure. The fact that the value by which the fracture decrease is bigger than the collapse pressure increase indicates that the permeability coefficient influences the fracture pressure more. Consider

(16) $$\begin{array}{c} \delta =\frac{\left(p_{w}-p_{o}\right)}{\left(p-p_{o}\right)},\;0\leq \delta \leq 1. \end{array}$$

#### 3.3.4. Stability in Deviated Borehole

**

**Figure 23:**
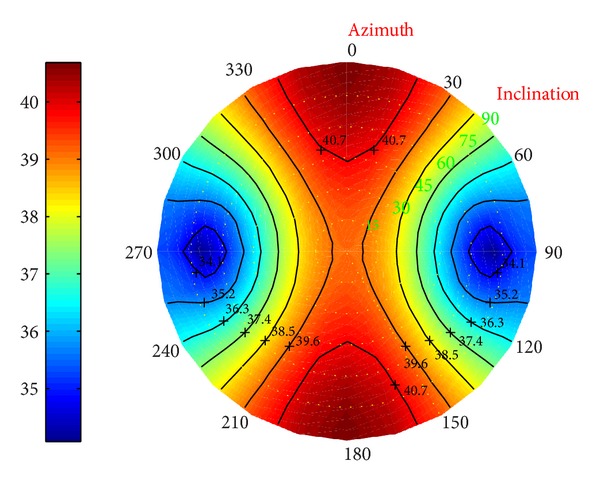
Risk distribution of collapse pressure when permeability coefficient is 0.5.

**Figure 24:**
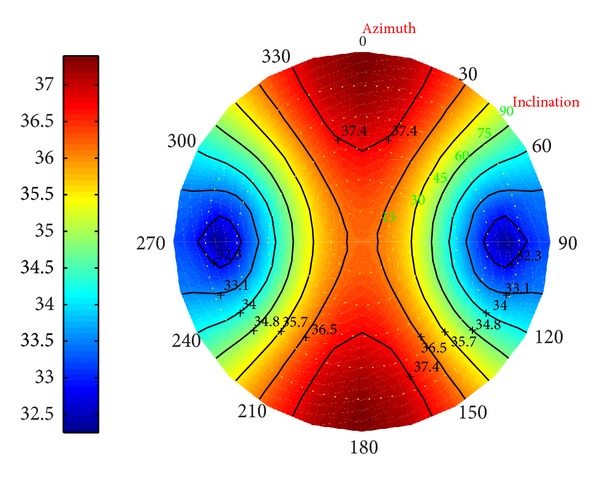
Risk distribution of collapse pressure when temperature drop is 25°C.

**Figure 25:**
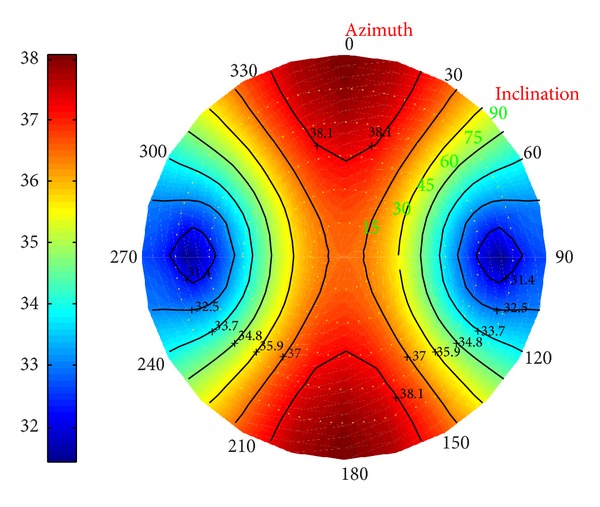
Risk distribution of collapse pressure under coupling of thermo-fluid-solid.

*(I) Collapse Pressure in Deviated Borehole*. Under different conditions, in Figures 23, 24, and 25 the distributions of the collapse pressure were performed.

Suppose north-south and east-west as the directions along which the horizontal maximum and minimum stresses developed, respectively. It can be concluded that the seepage can cut the maximum and add the minimum collapse pressures; the decrease of the temperature, however, leads to the increase of the maximum and minimum collapse pressure. What is more, Figure 25 shows that the minimum collapse pressure can reach the smallest, with the maximum ranking the middle. Without considering the fluid, the result will be below the prediction; on the other hand, without considering the temperature, the result will be beyond the prediction.

**Figure 26:**
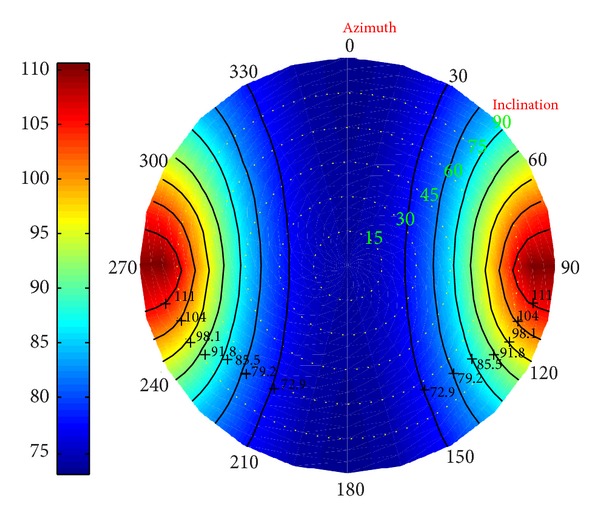
Risk distribution of fracture pressure when permeability coefficient is 0.5.

**Figure 27:**
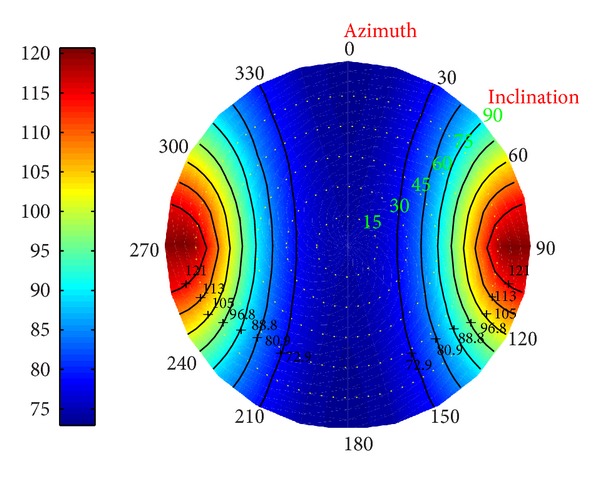
Risk distribution of fracture pressure when temperature drop is 25°C.

**Figure 28:**
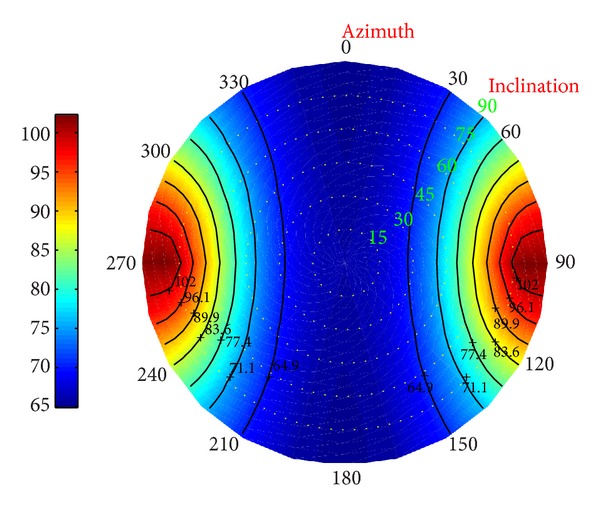
Risk distribution of fracture pressure under coupling of thermo-fluid-solid.

*(II) Fracture Pressure in Deviated Borehole.* Additionally, the distribution of the fracture pressure was performed in Figures 26, 27, and 28.

When drilling along the direction of the minimum principal stress, the fracture pressure reached the biggest, increasing the upper boundary of the fluid's density. It shows that the wider the window of the fluid is, the safer the drilling is. When drilling along the direction of the maximum stress, the fracture pressure reaches the minimum. As a result, it is suggested that in order to ensure the borehole stability, we should drill along the direction of the maximum stress. If the fracture pressure is beyond the expectation, the sloughing formation will be developed.

## 4. Conclusion

1. It is shown that the threshold temperature of strength and elastic modulus of granite are both 200 centigrade. Below this, the sample mainly undergoes the brittle fracture and the rupture surface is along the axial direction under small confining pressure, while shear compression failure is the main state when the confining pressure is over 20 MPa. Above 200 centigrade, the damage modes are mixing shear compression and brittle fracture failure, and shear compression failure is positively correlated with the increasing of confining pressure and temperature.
2. The compressional wave velocity, elastic modulus, and uniaxial compression strength will decrease as the temperature rises. Additionally, when the temperature is given, the elastic modulus and strength will increase as the surrounding pressure rises. The threshold pressure and temperature are 15 MPa and 200°C, respectively. The threshold thermal fracture temperature is 200°C. The permeability will dramatically increase with the rise of temperature up to 10^−3^~10^−4^ mD.
3. The coupling borehole stability model of thermo-fluid-solid is developed by the ANASYS-APDL. The dynamic evolution equations of elastic modulus, Poisson ratio, uniaxial compressive strength, and permeability of granite with temperature are built and run. The results show that the radical stress and tangential stress are greatly different in full coupling model and in other physical field models. The results simulated by full coupling model are more precise and reliable than other models.
4. The temperature affects the fracture pressure more than the collapse pressure. In order to avoid losing fluid, we suggest lowering the fluid's density when the temperature of the borehole wall decreases. As for the permeability, its rise leads to the decrease of the fracture pressure but increase of the collapse pressure, which indicates that the low-density fluid is better.
5. The seepage degrades the upper limit of collapse pressure and heightens the lower limit. The fall of temperature heightens both upper and lower limits of collapse pressure in borehole. As a result, in order to accurately predict the collapse pressure, the seepage and temperature are supposed to be taken into account.

## Nomenclature

*D*(*T*): Thermal damage coefficient
*T*: Temperature, °C
*E*_(*T*)_: Elastic modulus at *T*°C, GPa
*E*_(0)_: Elastic modulus at 20°C, GPa
*E*: Elastic modulus, GPa
*R*: Goodness of fit
*σ*_*s*_: Triaxial compressive strength, MPa
*σ*_*w*_: Confining pressure, MPa
*σ*′: Matrix of effective stress, MPa
*σ*: Matrix of total stress, MPa
*I*: Second-order unit tensor
*p*_*w*_: Absolute value of pressure, MPa
*n*: Porosity
*k*_1*ij*_: Permeability coefficient of fluid
*k*_1*T**ij*_: Velocity of flow coefficient affected by temperature
*μ*_1_: Viscosity coefficient of fluid
*ρ*_1_: Density of fluid, kg/m^3^
*p*_1_: Hydraulic pressure, Pa
*g*_*j*_: Acceleration of gravity of fluid, m/s^2^
*C*_*s*_: Specific heat capacity, J/kg · K
*ρ*_*s*_: Density of rock, kg/m^3^
*q*_*si*_: Heat flux density of rock, J/m^2^ · s
*Q*_*s*_: Energy conversion coefficient, J/m^3^ · s
*C*_1_: Specific heat capacity of fluid, J/kg · K
*v*_*li*_^*r*^: Relative density of fluid
*q*_*li*_^*c*^: Heat flow, W/m^2^
*q*_*mi*_: Total heat flux density, J/m^2^ · s
*q*_1*i*_: Heat flux density of fluid, J/m^2^ · s
*λ*_*si**j*_: Heat transfer coefficient of rock, W/m^2^ · K
*λ*_1*ij*_: Heat transfer coefficient of fluid, W/m^2^ · K
*λ*_*mi**j*_: Equivalent thermal conductivity coefficient, W/m^2^ · K
*ε*: Strain
*b*: Three-dimensional force, N
*t*: Plane vector force, N
*δ*: Cake permeability
*υ*: Poisson ratio
UCS: Uniaxial compressive strength, MPa
*K*: Permeability, mD
*σ*_1_: Maximum main stress, MPa
*σ*_3_: Minimum main stress, MPa
*φ*: Internal friction angle, rad
*C*: Cohesive force, N
*σ*_*θ*_: Tangential effective stress in borehole, MPa
*α*: Effective stress coefficient
*P*_*P*_: Pore pressure, MPa
*S*_*t*_: Tensile strength, MPa
*σ*_*c*_: Uniaxial compressive strength, MPa
*p*: Drilling fluid column pressure, MPa
*p*_*w*_: Borehole pore pressure, MPa
*p*_0_: Formation pore pressure.
